# Retrospective Study on the Application of Enhanced Recovery After Surgery Measures to Promote Postoperative Rehabilitation in 50 Patients With Brain Tumor Undergoing Craniotomy

**DOI:** 10.3389/fonc.2021.755378

**Published:** 2021-11-12

**Authors:** SongShan Feng, Bo Xie, ZhenYan Li, XiaoXi Zhou, Quan Cheng, ZhiXiong Liu, ZiRong Tao, MingYu Zhang

**Affiliations:** ^1^ Department of Neurosurgery, Xiangya Hospital, Central South University, Changsha, China; ^2^ National Clinical Research Center for Geriatric Disorders, Xiangya Hospital, Central South University, Changsha, China; ^3^ Teaching and Research Section of Clinical Nursing, Xiangya Hospital, Central South University, Changsha, China

**Keywords:** brain tumor, ERAS, craniotomy, postoperative rehabilitation, prognosis

## Abstract

**Objective:**

To investigate whether enhanced recovery after surgery (ERAS) can promote rehabilitation of patients after neurosurgical craniotomy.

**Methods:**

The clinical data of 100 patients with brain tumor undergoing craniotomy in the Department of Neurosurgery, Xiangya Hospital, Central South University, from January 2018 to August 2020 were collected, including 50 patients in the ERAS group and 50 patients in the control group. t-Test, Wilcoxon’s rank sum test, and chi-square analysis were used to compare the clinical characteristics, prognosis, and hospitalization time between the two groups.

**Results:**

There was no significant difference in gender, age, and other general clinical data between the two groups (*p* > 0.05). The days of antiemetic drugs applied in the ERAS group were less than those in the control group (1.00 *vs*. 2.00 days, *p* = 0.003), and the proportion of patients requiring analgesics was lower than that of the control group (30% *vs*. 52%, OR = 0.41, 95% CI 0.18–0.93, *p* = 0.031). The time of urinary catheter removal and that of patients starting ambulation in the ERAS group were shorter than those in the control group (16.00 *vs*. 24.00 h, and 1.00 *vs*. 2.00 days, *p* < 0.001, respectively); and the hospital length of stay (LOS) in the ERAS group was shorter than that in the control group (Total LOS, 13.00 *vs*. 15.50 days; Postoperative LOS, 7.00 *vs*. 10.00 days, *p* < 0.001). By analyzing the prognosis of patients in the ERAS group and control group, we found that there was no significant difference in postoperative complications and Karnofsky Performance Status (KPS) score 1 month after operation between the two groups.

**Conclusion:**

The application of ERAS in craniotomy can accelerate the postoperative recovery of patients without increasing the perioperative risk, which is worthy of wide application. However, whether the ERAS measures can reduce the postoperative complications and improve the prognosis of patients still needs more large-scale case validation and multicenter collaborative study.

## 1 Introduction

The concept of enhanced recovery after surgery (ERAS) was first proposed by Professor Kehlet of Denmark (1), which refers to the adoption of a series of perioperative optimization measures supported by evidence-based medical evidence to accelerate patients’ rehabilitation and reduce postoperative complications. Surgeons had achieved remarkable results in shortening hospital stay and reducing hospital expenses by applying the ERAS protocols (2). ERAS has been popularized and applied in many surgical fields, including general surgery and orthopedics. In recent years, the European ERAS society has continuously issued ERAS operation guidelines for many disciplines (3–5). Previously, it was believed that neurosurgical craniotomy required a number of invasive monitoring methods, and most postoperative patients needed to enter intensive care unit and recovered slowly (6). Therefore, there are few reports on the application of ERAS in neurosurgery. However, with the development of microsurgical technique, the continuous progress of multimodal imaging, neuro-navigation, brain function monitoring and other technologies, and the deepening of multidisciplinary cooperation, the postoperative recovery time of patients undergoing craniotomy has been greatly shortened (7). Hagan et al. proposed 17 accelerated rehabilitation measures for craniotomy in 2016, including preoperative counseling, minimally invasive craniotomies, and postoperative artificial nutrition (8). Wang et al. reported a prospective randomized controlled study on the application of accelerated rehabilitation measures in craniotomy in 2018. They believe that ERAS can shorten the hospital length of stay (LOS) and does not increase the perioperative risk (9). However, the ERAS system for craniotomy that can be popularized and applied on a large scale is still limited. This study retrospectively analyzed the clinical data of 100 patients with brain tumors who underwent craniotomy in the Department of Neurosurgery of Xiangya Hospital from January 2018 to August 2020, explored the promoting effect of the ERAS measures on postoperative rehabilitation, and provided evidence for the establishment of the ERAS system for patients with brain tumors accepting craniotomy.

## 2 Materials and Methods

### 2.1 Patient Recruitment

This study collected the clinical data of 100 patients with brain tumor hospitalized in the Department of Neurosurgery of Xiangya Hospital from January 2018 to August 2020, including 50 patients accepting accelerated rehabilitation treatment (ERAS group) and 50 patients accepting routine surgical treatment (Control group). The inclusion criteria were as follows: 1) 18–65 years old; 2) preoperative diagnosis was brain tumor; 3) there was no obvious neurological and cognitive impairment before operation; and 4) there were no serious concomitant diseases that may affect the prognosis (such as heart failure and chronic renal insufficiency).

The general clinical data collected included patient name, hospitalization number, gender, age, body mass index (BMI), concomitant disease, smoking and drinking history, and preoperative albumin value; the tumor pathology, tumor location, tumor size, blood loss during surgery, and intraoperative blood transfusion; and hospital LOS, hospitalization expenses, postoperative complications, and Karnofsky Performance Status (KPS) score.

### 2.2 Enhanced Recovery After Surgery Protocol and Clinical Data Collection

The accelerated rehabilitation measures taken by the ERAS group include the following: 1) preoperative evaluation of vomiting risk and application of antiemetics to prevent postoperative vomiting was performed; 2) 200 ml of carbohydrates was taken orally 2 h before operation, and oral feeding was resumed as early as possible after operation; 3) preoperative thrombosis risk assessment and postoperative thrombosis prevention were performed; 4) scalp nerve block and local infiltration anesthesia with ropivacaine were used to reduce postoperative pain; 5) indwelling wound drainage tube during operation was avoided; 6) the urinary catheter was removed as soon as possible after operation; and 7) early ambulation training was performed with the help of nurses after operation. The perioperative management of patients in the control group was mainly based on the personal experience of surgeons and anesthesiologists in our institution.

The data collected included preoperative postoperative nausea and vomiting (PONV) score, preoperative PONV prophylaxis, and the days of postoperative use of antiemetic drugs; postoperative feeding time, proton pump inhibitor application time, and intravenous infusion volume; preoperative thrombosis risk assessment (Caprini score), postoperative thrombosis prophylaxis measures, and whether venous thrombosis occurred; postoperative pain assessment applying Visual Analogue Scale (VAS) score and whether analgesics were used; whether the wound drainage tube was retained during the operation; urinary catheter removal time after surgery; and ambulation time after surgery.

### 2.3 Compliance With Ethical Standards

The study was approved by the Ethics Committee of Xiangya Hospital (Ethics approval No. 2018111102). All participants signed written informed consent.

### 2.4 Statistical Analysis

The measurement data conforming to the normal distribution are represented by “mean ± SD,” and the measurement data not conforming to the normal distribution are represented by “median (interquartile spacing).” Student’s t-test was used for the comparison between the two groups of measurement data conforming to normal distribution, and Wilcoxon’s rank sum test was used for the comparison between that not conforming to normal distribution. Categorical data were described by rate, and chi-square test was used for inter-group comparison. *p* < 0.05 was considered statistically significant. SPSS 25.0 (IBM Corp.) was used for data statistics and analysis.

## 3 Results

### 3.1 Characteristics of Clinical Data

Fifty patients were included in the ERAS group and control group. The average ages of the two groups were 48.12 ± 11.89 and 48.10 ± 15.26 years, respectively (*p* = 0.994). There were 29 female cases (58%) in the ERAS group and 27 female cases (54%) in the control group (*p* = 0.687). There was no significant difference in BMI, concomitant diseases (including cardiovascular system, respiratory system, digestive system, diabetes, and multiple complications), smoking history, drinking history, and preoperative serum albumin level between the two groups (**Table 1**).

**Table 1:** Clinical characteristics of 100 patients who underwent craniotomy.

	ERAS group	Control group	*p*-Value
No. of patients	50	50	
Average age (years)	48.12 ± 11.89	48.10 ± 15.26	0.994
Gender			0.687
Male, no. (%)	21 (42%)	23 (46%)	
Female, no. (%)	29 (58%)	27 (54%)	
Average BMI value	23.28 ± 3.26	22.79 ± 2.89	0.429
Concomitant disease, no. (%)			
Cardiovascular system	8 (16%)	6 (12%)	0.564
Respiratory system	1 (2%)	2 (4%)	0.558
Digestive system	4 (8%)	4 (8%)	1.000
Diabetes	4 (8%)	3 (6%)	0.695
Multiple (≥2 systems)	2 (4%)	1 (2%)	0.558
Smoking history, no. (%)	6 (12%)	4 (8%)	0.505
Drinking history, no. (%)	3 (6%)	1 (2%)	0.307
Preoperative albumin value (g)	41.25 ± 2.92	40.91 ± 3.35	0.583

ERAS, enhanced recovery after surgery; BMI, body mass index.

Patients in both groups accepted craniotomy. There was no significant difference in tumor characteristics between the two groups (**Table 2**). The tumor pathological diagnosis was mainly meningioma and glioma. There were 13 cases (26%) diagnosed as glioma in the ERAS group and 18 cases (36%) in the control group (*p* = 0.280). For meningioma, the data were 16 cases (32%) and 15 cases (30%), respectively (*p* = 0.829). The tumors were mainly supratentorial. There were 45 supratentorial cases (90%) in the ERAS group and 46 supratentorial cases (92%) in the control group (*p* = 0.727). The maximum tumor diameter of the two groups was 3.65 (2.63) and 3.00 (2.00) cm (*p* = 0.453). The median blood loss in both groups was 200.00 ml (*p* = 0.261), and the number of blood transfusion patients was four cases (8%) and five cases (10%), respectively (*p* = 0.727).

**Table 2:** Summary of tumor and operation related details.

	ERAS group	Control group	*p*-Value
Tumor pathology			
Glioma, no. (%)	13 (26%)	18 (36%)	0.280
Meningioma, no. (%)	16 (32%)	15 (30%)	0.829
Others, no. (%)	21 (42%)	17 (34%)	0.410
Lesion location			0.727
Supratentorial, no. (%)	45 (90%)	46 (92%)	
Subtentorial, no. (%)	5 (10%)	4 (8%)	
Maximum tumor diameter (cm) median (interquartile spacing)	3.6 (2.63)	3.00 (2.00)	0.453
Craniotomy, no. (%)	50 (100%)	50 (100%)	1.000
Median blood loss during surgery in ml median (interquartile spacing)	200.00 (162.50)	200.00 (200.00)	0.261
RBC transfusion during surgery, no. (%)	4 (8%)	5 (10%)	0.727

ERAS, enhanced recovery after surgery; RBC, red blood cell.

### 3.2 Measures and Effects of Enhanced Recovery After Surgery Protocols

#### 3.2.1 Preoperative Antiemetic Drugs Were Used to Prevent Postoperative Vomiting

PONV score was used to evaluate the risk of postoperative vomiting in the ERAS group and control group before operation. There was no significant difference in PONV score between the two groups (**Table 3**). The proportion of patients accepting PONV prophylaxis in the ERAS group was significantly higher than that in the control group (58% *vs*. 0%, *p* < 0.001), and the days of using antiemetics after operation were significantly less than those in the control group (1.00 *vs*. 2.00 days, *p* = 0.003).

**Table 3:** The first part of ERAS measures and effects.

	ERAS group	Control group	*p*-Value
1. Preoperative antiemetic drugs were used to prevent postoperative vomiting			
Preop PONV score			
0, no. (%)	4 (8%)	2 (4%)	0.400
1, no. (%)	17 (34%)	21 (42%)	0.410
2, no. (%)	29 (58%)	27 (54%)	0.687
Preop PONV prophylaxis, no. (%)	29 (58%)	0	<0.001
Days of using antiemetic drugs after operation			
Median (interquartile spacing)	1.00 (1.00)	2.00 (1.00)	0.003
2. 200 ml of carbohydrates was taken orally 2 h before operation, and oral feeding was resumed early after operation			
Preop oral carbohydrate, no. (%)	50 (100%)	0	<0.001
Postop diet Median (interquartile spacing)			
Postop water intake time (h)	6.00 (2.00)	6.00 (1.00)	0.001
Postop liquid food intake time (h)	12.00 (0)	14.00 (2.00)	<0.001
Postop solid food intake time (h)	24.00 (0)	25.00 (2.00)	<0.001
Postop application time of proton pump inhibitors (days)	1.00 (1.00)	2.00 (1.00)	<0.001
Postop intravenous infusion volume			
Median (interquartile spacing)			
Intravenous infusion volume on Postop day 1 (ml)	2,300.00 (400.00)	2,400.00 (300.00)	0.147
Intravenous infusion volume on Postop day 2 (ml)	800.00 (250.00)	1,200.00 (200.00)	<0.001
Intravenous infusion volume on Postop day 3 (ml)	375.00 (162.50)	600.00 (125.00)	<0.001

ERAS, enhanced recovery after surgery; PONV, postoperative nausea and vomiting.

#### 3.2.2 200 ml of Carbohydrates Was Taken Orally 2 h Before Operation, and Oral Feeding Was Resumed Early After Operation

All patients in the ERAS group took 200 ml of carbohydrates orally 2 h before operation and started oral feeding as soon as possible after operation. The patients in the control group followed routine fasting for 8 h before operation. The postoperative water intake time, liquid food intake time, and solid food intake time in the ERAS group were 6.00 (2.00), 12.00 (0), and 24.00 (0) h, respectively; and the corresponding time in the control group was 6.00 (1.00), 14.00 (2.00), and 25.00 (2.00) h, respectively, with *p*-values all less than 0.05. The application time of proton pump inhibitors in the two groups was 1.00 (1.00) and 2.00 (1.00) days (*p* < 0.001). On the first day, the second day, and the third day after operation, the intravenous infusion volume in the ERAS group was 2,300.00 (400.00), 800.00 (250.00), and 375.00 (162.50) ml, respectively; and the corresponding infusion volume in the control group was 2,400.00 (300.00), 1,200.00 (200.00), and 600.00 (125.00) ml, respectively, with *p*-values of 0.147, <0.001, and <0.001, respectively (**Table 3**).

#### 3.2.3 Preoperative Thrombosis Risk Assessment and Postoperative Thrombosis Prophylaxis

The risk of thrombosis was assessed by Caprini score before operation in both the ERAS group and control group, and there was no significant difference between the two groups. In the ERAS group, 19 patients (38%), 19 patients (38%), 10 patients (20%), and two patients (4%) were assessed as low, middle, high, and extremely high risk of venous thrombosis, respectively, while in the control group, 22 patients (44%), 19 patients (38%), seven patients (14%), and two patients (4%) were assessed, respectively, with *p*-values greater than 0.05 (**Table 4**). All patients in both groups were treated with lower limbs active/passive activities to prevent thrombosis (n = 50, 100%). The patients in the ERAS group concurrently received mechanical prevention (intermittent pneumatic compression) (n = 50, 100%), and patients in the control group did not receive mechanical prevention (*p* < 0.001). The patients with postoperative deep venous thrombosis in the two groups were one case (2%) and two cases (4%) (*p* = 0.558).

**Table 4:** The second part of ERAS measures and effects.

	ERAS group	Control group	*p*-Value
3. Preoperative thrombus risk assessment and postoperative thrombus prophylaxis			
Thrombus risk assessment			
Low risk (Caprini score 0–1), no. (%)	19 (38%)	22 (44%)	0.542
Middle risk (Caprini score 2), no. (%)	19 (38%)	19 (38%)	1.000
High risk (Caprini score 3–4), no. (%)	10 (20%)	7 (14%)	0.424
Extremely high risk (Caprini score ≥ 5), no. (%)	2 (4%)	2 (4%)	1.000
Lower limbs active/passive activity, no. (%)	50 (100%)	50 (100%)	
Mechanical prophylaxis (intermittent pneumatic compression), no. (%)	50 (100%)	0	<0.001
Patients developing thrombus postoperatively, no. (%)	1 (2%)	2 (4%)	0.558
4. Intraoperative scalp nerve block and local infiltration anesthesia to reduce postoperative pain			
Patients receiving intraoperative scalp nerve block and local infiltration anesthesia, no. (%)	50 (100%)	0	<0.001
Postoperative pain assessment			
Mild (VAS 0–3), no. (%)	38 (76%)	24 (48%)	0.004
Moderate (VAS 4–6), no. (%)	9 (18%)	19 (38%)	0.026
Severe (VAS 7–10), no. (%)	3 (6%)	7 (14%)	0.182
Patients receiving postoperative analgesic drugs, no. (%)	15 (30%)	26 (52%)	0.031

ERAS, enhanced recovery after surgery; VAS, Visual Analogue Scale.

#### 3.2.4 Intraoperative Scalp Nerve Block and Local Infiltration Anesthesia Were Applied to Reduce Postoperative Pain

The intravenous anesthetics and inhaled anesthetics were the same in the two groups. Patients in the ERAS group were treated with scalp nerve block and local infiltration anesthesia with ropivacaine at surgical incision to reduce postoperative pain (n = 50, 100%), and patients in the control group did not accept this handling (**Table 4**). The number of patients with postoperative mild pain (VAS 0–3), moderate pain (VAS 4–6), and severe pain (VAS 7–10) in the ERAS group was 38 (76%), 9 (18%), and 3 (6%); and the corresponding number of patients in the control group was 24 (48%), 19 (38%), and 7 (14%). *p*-Values were 0.004, 0.026, and 0.182, respectively. The proportion of patients receiving postoperative analgesic drugs in the ERAS group was significantly lower than that in the control group (30% *vs*. 52%, OR = 0.41, 95% CI 0.18–0.93, *p* = 0.031).

#### 3.2.5 Other Enhanced Recovery After Surgery Protocols

Other ERAS protocols include avoiding indwelling wound drainage tube during operation, removing urinary catheter as soon as possible, and early ambulation training with the help of nurses after operation (**Table 5**). In this study, there was only one patient (2%) accepting wound drainage tube in both the ERAS group and control group. The median time of urinary catheter removal after surgery in the ERAS group was 16.00 (12.00) h, and the time was 24.00 (2.25) h in the control group (*p* < 0.001). The median time of patients in the ERAS group starting to ambulate after surgery was 1.00 (1.00) day, and the time was 2.00 (1.25) days in the control group (*p* < 0.001).

**Table 5:** The third part of ERAS measures and effects.

	ERAS group	Control group	*p*-Value
5. Avoiding indwelling wound drainage tube during operation			
Patient accepting wound drainage tube, no. (%)	1 (2%)	1 (2%)	1.000
6. Remove the urinary catheter as soon as possible after operation			
Median time of urinary catheter removal after surgery (h)	16.00 (12.00)	24.00 (2.25)	<0.001
7. Early ambulation after operation			
The median time to ambulate after surgery (days)	1.00 (1.00)	2.00 (1.25)	0.001

ERAS, enhanced recovery after surgery.

### 3.3 Hospital Length of Stay and Expenses

The total hospital LOS and postoperative LOS in the ERAS group were 13.00 (3.00) and 7.00 (1.00) days, respectively; and the corresponding time in the control group was 15.50 (3.00) and 10.00 (3.00) days (*p* < 0.001). The hospitalization expenses of the ERAS group and control group were 58,146.35 (7,688.28) and 64,815.91 (12,257.06) yuan, respectively (*p* < 0.001) (**Table 6**).

**Table 6:** Analysis of median hospital LOS, cost of hospitalization, and prognosis of patients.

	ERAS group	Control group	*p*-Value
Hospital LOS median (interquartile spacing)			
Total LOS (days)	13.00 (3.00)	15.50 (3.00)	<0.001
Postop LOS (days)	7.00 (1.00)	10.00 (3.00)	<0.001
Cost of hospitalization (yuan) median (interquartile spacing)	58,146.35 (7,688.28)	64,815.91 (12,257.06)	<0.001
Intracranial complications, no. (%)			
Intracranial infection	1 (2%)	2 (4%)	0.558
Neurological dysfunction	2 (4%)	2 (4%)	1.000
Epilepsy	1 (2%)	1 (2%)	1.000
Systemic complications, no. (%)			
Pulmonary infection	2 (4%)	2 (4%)	1.000
Deep venous thrombosis	1 (2%)	2 (4%)	0.558
1-month follow-up KPS score after surgery			0.727
≥80, no. (%)	46 (92%)	45 (90%)	
50–70, no. (%)	4 (8%)	5 (10%)	

LOS, length of stay; ERAS, enhanced recovery after surgery; KPS, Karnofsky Performance Status.

### 3.4 Postoperative Complications and Patient Prognosis

The postoperative complications include intracranial complications and systemic complications. The complications caused by intracranial factors in the ERAS group included one case of intracranial infection (2%), two cases of neurological dysfunction (4%), and one case of epilepsy (2%). The corresponding complications in the control group were two cases of intracranial infection (4%), two cases of neurological dysfunction (4%), and one case of epilepsy (2%). The *p*-values were all greater than 0.05 (**Table 6**). The systemic complications in the ERAS group included two cases of pulmonary infection (4%) and one case of lower extremity deep venous thrombosis (2%), and the corresponding complications in the control group were two cases of pulmonary infection (4%) and two cases of lower extremity deep venous thrombosis (4%). The *p*-values were both greater than 0.05. The 1-month follow-up data after surgery showed that the number of patients with KPS score ≥80 in the ERAS group was 46 (92%), the number of patients with a score of 50–70 was 4 (8%), and the corresponding number of patients in the control group was 45 (90%) and five cases (10%) (*p* = 0.727).

## 4 Discussion

The implementation of ERAS in multiple surgeries has been proved to shorten hospitalization time, reduce hospitalization expenses, and accelerate rehabilitation of patients, but it is rarely used in neurosurgery. We summarized the ERAS protocols and outcome measures applied in elective craniotomy in the past studies (**Table 7**). Wang et al. reported a prospective randomized controlled study on the application of the ERAS measures in craniotomy in 2018, and they proposed a multidisciplinary management process from preoperative evaluation, intraoperative management to postoperative rehabilitation measures. The conclusion shows that the application of the ERAS measures in patients undergoing elective craniotomy can accelerate the rehabilitation of patients with safety and effectiveness (9). The concept of ERAS emphasizes to minimize the patient’s stress response and restore patient’s normal physiological functions as soon as possible. Therefore, this study put forward seven important ERAS measures based on this concept, which aim to reduce postoperative stress reactions such as pain and vomiting and to restore normal physiological functions such as oral eating and ambulation as soon as possible, so as to accelerate rehabilitation of patients.

**Table 7:** Summarization of the ERAS protocols and outcome measures applied in elective craniotomy in the past studies.

Authors	Study design	Key ERAS protocols	Outcome measures
Yuan Wang et al. (9)	Prospective randomized controlled trial	PONV management, preop fasting and carb loading, scalp incision anesthetic with ropivacaine, intravenous antibiotics given before incision, surgical incision suturing, evaluation and prophylactic antithrombotic therapy, urinary drainage, postop diet, adherence to ambulation	Median total hospital LOS from admission to discharge, median hospital LOS from end of procedure to discharge, 30-day all-cause readmission rate, reoperation rate for any indication within 30 days, total cost of hospitalization in RMB, surgical complication, nonsurgical complication, functional recovery
Anirudh Elayat et al. (10)	Non-randomized controlled trial	Family education, complex-carbohydrate drink, flupirtine, scalp blocks, limited opioids, rigorous fluid and temperature regulation, flupirtine, early mobilization, removal of catheters, initiation of feeds	Length of ICU stay, pain scores in ICU, opioid requirement, glycemic control, hospital stay duration
Katherine B. Hagan et al. (8)	Literature review	Preoperative counseling, preoperative smoking and alcohol consumption, preoperative enteral nutrition and perioperative oral immunonutrition, preoperative fasting and carbohydrate loading, anti-thrombotic prophylaxis, antimicrobial prophylaxis and skin preparation, scalp blocks, anesthetic protocol, non-opioid analgesia, PONV, minimally invasive craniotomies and endoscopic skull base approaches, avoiding hypothermia, fluid balance, urinary drainage, postoperative artificial nutrition, early mobilization, audit	NA

ERAS, enhanced recovery after surgery; LOS, length of stay; ICU, ICU, intensive care unit; PONV, postoperative nausea and vomiting. NA, Not Available.

PONV are common adverse reactions, which will delay the recovery of patients (11). For patients undergoing craniotomy, postoperative vomiting may increase intracranial pressure and cause serious complications such as brain edema and intracerebral hemorrhage. Therefore, we evaluated the risk of PONV by Apfel adult PONV risk score (12), and we applied preventive measures for patients with a score ≥2. The preventive drug is the combination of dexamethasone and serotonin receptor antagonist (8, 9). In this study, 29 patients in the ERAS group were treated with preventive measures (58%) before operation, and the median antiemetic drug application time after operation was 1.00 day. There were no patients receiving preventive measures in the control group according to the traditional processing method, and the median antiemetic drug application time after operation was 2.00 days, which was significantly longer than that in the ERAS group (*p* = 0.003).

Previous studies have shown that compared with long-term fasting before surgery, oral carbohydrate 2 h before surgery can reduce patients’ insulin resistance; improve patients’ subjective feelings of thirst, hunger, and fatigue after operation; and do not increase the occurrence of postoperative vomiting (8, 13). In this study, all patients in the ERAS group took 200 ml of carbohydrates orally 2 h before surgery, and there was no aspiration or vomiting during operation. Except for patients with consciousness disorder after operation, craniotomy generally does not affect the digestive tract function of patients. Therefore, we encourage patients to resume oral feeding early after operation and to reduce the amount of intravenous infusion and the use of proton pump inhibitors. Our results show that this measure can quickly restore the postoperative gastrointestinal function and accelerate the perioperative rehabilitation.

The prevention of deep venous thrombosis in the perioperative period of craniotomy is mainly based on mechanical prevention, but drug prevention can also be considered when the risk of bleeding is low (14). The mechanical prevention is applied as the ERAS measure previously, including intermittent pneumatic compression and graduated compression stockings (8, 9). All patients in this study received lower limb active/passive activities to prevent thrombosis, but only patients in the ERAS group received mechanical prevention concurrently (intermittent pneumatic compression). In the study conducted by Wang, there was no patient suffering from deep venous thrombosis in the ERAS group, while there were two patients suffering from deep venous thrombosis in the control group (3%). Although the *p*-value was greater than 0.05, the authors believed that mechanical prevention could effectively reduce the incidence of perioperative deep venous thrombosis (9). In our study, the numbers of patient developing postoperative deep venous thrombosis in the ERAS group and control group were one (2%) and two (4%), respectively; and the *p*-value was also greater than 0.05. However, we believe that it may be due to the small sample size and because perioperative mechanical prevention can effectively reduce the formation of deep venous thrombosis.

Pain management of craniotomy is an important part of the ERAS process. Reducing postoperative pain can accelerate rehabilitation and improve comfort and satisfaction of patients. Scalp nerve block and incision infiltration anesthesia can effectively reduce postoperative pain and the use of analgesic drugs (8, 15), which are implemented by anesthesiologists and surgeons, respectively. Qu et al. showed that scalp infiltration anesthesia with ropivacaine in the ERAS group could effectively reduce the degree and time of postoperative pain (16). In our study, all patients in the ERAS group were treated with scalp nerve block and infiltration anesthesia before scalp incision. The results showed that the degree of pain was significantly lower and the application of analgesic drugs was less in the ERAS group than in the control group.

Prolonged indwelling of urinary catheter after operation may lead to urinary tract infection and may restrict patient’s mobilization (17). Therefore, we emphasize early removal of urinary catheter after operation to restore normal physiological function as soon as possible. In this study, the urinary catheter was removed about 16 h after operation in the ERAS group, and there were no patients developing urinary tract infection. It has been reported that early postoperative ambulation can improve patients’ cardiopulmonary function and reduce the incidence of postoperative venous thrombosis (18, 19). In this study, patients in the ERAS group began to ambulate on the first day after operation, which we believe can accelerate the process of postoperative rehabilitation.

By comparing and analyzing the prognosis of patients in the ERAS group and control group, we found that there was no significant difference in surgical complications and KPS score at 1 month after surgery between the two groups. We believe that it may be related to the small sample size, and ERAS measures can reduce postoperative complications and improve prognosis of patients, which is consistent with the conclusion of Wang’s research (9). In addition, the results showed that the total hospital LOS, postoperative LOS, and hospitalization expenses of the ERAS group were significantly lower than those of the control group, suggesting the effectiveness and economic benefits of the ERAS measures.

## 5 Conclusion

Here, we provide our experience in the application of ERAS in craniotomy. We believe that the application of the ERAS measures in craniotomy can accelerate the postoperative rehabilitation of patients without additional perioperative risk, which is worthy of widespread promotion and application. However, whether the ERAS measures can reduce postoperative complications and improve patients’ prognosis requires more large-scale case validation and multicenter collaborative research.

## Data Availability Statement

The original contributions presented in the study are included in the article/supplementary material. Further inquiries can be directed to the corresponding authors.

## Ethics Statement

The studies involving human participants were reviewed and approved by Medical Ethics Committee of Xiangya Hospital. The patients/participants provided their written informed consent to participate in this study.

## Author Contributions

SSF, BX, QC, and XXZ prepared the manuscript, analyzed the data, and performed the experiments. ZYL and ZXL designed the experiments. MYZ modified the manuscript. ZRT and MYZ designed the project and finally approved the manuscript to be published. All authors contributed to the article and approved the submitted version.

## Funding

This work was supported by the Hunan Provincial Natural Science Foundation of China (Grant No. 2020JJ8111) and Clinical Medical Technology Innovation Guidance Program of Hunan Province (No. 2017SK50109).

## Conflict of Interest

The authors declare that the research was conducted in the absence of any commercial or financial relationships that could be construed as a potential conflict of interest.

## Publisher’s Note

All claims expressed in this article are solely those of the authors and do not necessarily represent those of their affiliated organizations, or those of the publisher, the editors and the reviewers. Any product that may be evaluated in this article, or claim that may be made by its manufacturer, is not guaranteed or endorsed by the publisher.
